# Occurrence of BNT162b2 Vaccine Adverse Reactions Is Associated with Enhanced SARS-CoV-2 IgG Antibody Response

**DOI:** 10.3390/vaccines9090977

**Published:** 2021-09-01

**Authors:** Yoav Rechavi, Moshe Shashar, Jonathan Lellouche, Moshe Yana, Daniel Yakubovich, Nechama Sharon

**Affiliations:** 1 Sackler Faculty of Medicine, Tel-Aviv University, Tel-Aviv 6997801, Israel; 2 Ruth and Bruce Rappaport Faculty of Medicine, Technion, Haifa 3525433, Israel; mshashar@laniado.org.il; 3 Nephrology Section, Laniado Hospital, Netanya 4244916, Israel; 4 Clinical Laboratories Department, Laniado Hospital, Netanya 4244916, Israel; jlellouche@laniado.org.il; 5 Pediatric Hemato-Oncology Department, Laniado Hospital, Netanya 4244916, Israel; myana@laniado.org.il (M.Y.); nsharon@laniado.org.il (N.S.); 6 Department of Neonatology, Schneider Children’s Hospital, Petach Tikva 4920235, Israel; danial@tauex.tau.ac.il; 7 Preterm Follow-Up Clinic, Sanz Medical Center, Laniado Hospital, Netanya 4244916, Israel

**Keywords:** COVID-19, SARS-CoV-2, vaccine, vaccination hesitancy, BNT162b2, antibodies, adverse reactions

## Abstract

Promoting SARS-CoV-2 vaccination has been a global mission since the first vaccines were approved for emergency use. Alongside the excitement following the possibility of eradicating SARS-CoV-2 and ending the COVID-19 pandemic, there has been ample vaccine hesitancy, some due to the abundant reporting of adverse reactions. We report here that the occurrence of BNT162b2 vaccine adverse reactions is associated with enhanced antibody response. We found a statistically significant correlation between having an adverse reaction, whether local or systemic, and higher antibody levels. No sex difference was observed in antibody levels. However, as was recently reported, the antibody response was found to be lower among older vaccinees. The demonstration of a clear correlation between adverse reactions and antibody levels may help reduce vaccination hesitancy by reassuring that the presence of such reactions is an indication of a well-functioning immune system.

## 1. Introduction

COVID-19, caused by the infection of SARS-CoV-2, was declared a pandemic by the World Health Organization in March 2020. Since then, nearly 200 million cases and more than 4 million related deaths have been confirmed [1]. In December 2020, the first FDA emergency use authorization was granted for the BNT162b2 vaccine, an mRNA-based vaccine for the prevention of SARS-CoV-2 infection and disease, followed by approval of additional vaccines.

In order to contain the pandemic and minimize COVID-19 morbidity and mortality, a large portion of the population must acquire resistance to SARS-CoV-2, either by vaccination or through exposure to the native virus, preferably the former. The development, implementation, and distribution of safe and effective vaccines marked a turning point in the global effort to restrain the pandemic, as some countries managed to, at least temporarily, return to pre-COVID lives with fast and efficient mass vaccination delivery.

Unfortunately, even countries with a wide availability of vaccines seem to encounter difficulties reaching sufficient containment, mainly due to vaccination hesitancy and the emergence of more elusive, infective variants (such as the Delta variant, also known as lineage B.1.617.2).

To this date, more than 4 billion vaccine doses have been administered worldwide [1], many of them mRNA-based vaccines. The occurrence of serious adverse events seen thus far is extremely rare, as opposed to the proven mortality and morbidity, both short and long term, of the COVID-19 disease [2,3,4,5,6]. Despite the clear evidence that vaccination is beneficial, vaccination hesitancy is a major obstacle to overcoming the pandemic, fueled by anti-vaccination movements and exaggerated media coverage and social media exposure [7,8]. In addition to “traditional” vaccines reluctance, the implementation of novel vaccine types based on synthetic mRNA further enhanced the resistance to vaccination based on unproven and speculative claims, which are not supported by biological evidence and the large experience gained over the past year.

We provide here our data, indicating that the occurrence of common, non-serious adverse reactions to the BNT162b2 vaccine is associated with increased antibody response. This information may be relevant to future decisions regarding vaccination policies worldwide and can help alleviate vaccination hesitancy.

## 2. Materials and Methods

### 2.1. Study Population

All fully vaccinated healthcare workers of one hospital, the Sanz Medical Center Laniado Hospital, were asked to fill an online questionnaire. All responders were included in the study. One individual was previously diagnosed with a COVID-19 infection and was excluded. The study was approved by the Laniado Hospital institutional review board (IRB), approval number: 0007-21-LND.

### 2.2. Antibodies Quantification

SARS-CoV-2 IgG antibodies were quantified using a chemiluminescent microparticle immunoassay (SARS-CoV-2 IgG II Quant on an ARCHITECT analyzer, Abbott, North Chicago, IL, USA) in vaccinees plasma. The assay principle is based on the detection of IgG antibodies, including neutralizing antibodies, to the receptor-binding protein of the S1 subunit of the spike protein of SARS-CoV-2, with a reported positive predictive agreement of 99.4% (CI 96.50% to 99.97%), a negative predictive agreement of 99.6% (CI 99.15% to 99.37%), and in agreement with a neutralization method (positive agreement, 100.0%; CI 95.72% to 100.00%) [9]. An antibody response with a value of ≥50 AU/mL was defined as a positive response by the assay’s manufacture.

### 2.3. Adverse Reactions Documentation

The fully vaccinated healthcare workers were asked to fill an online questionnaire regarding BNT162b2 COVID-19 vaccine adverse reactions following the administration of the 2 vaccine doses. The questionnaire included questions regarding their age, occupation, adverse reactions after each vaccine dose, date of vaccination (both doses), underlying medical conditions, and medications. Local and systemic reactions were categorized according to Polack et al. [10], where injection site pain, redness, and swelling were classified as local, and any other adverse reactions were classified as systemic.

### 2.4. Statistics

Statistical analyses were performed using Sigmaplot 11 (Systat software Inc., San Jose, CA, USA).

## 3. Results

The early institution of BNT162b2 COVID-19 vaccination among Israeli healthcare workers allowed us to analyze the association between vaccine adverse reactions and antibody response. We drew blood from 136 fully immunized healthcare workers on an average of 32.4 ± 4.84 days following the second vaccine dose and quantified their antibody response. The fully vaccinated healthcare workers were asked to fill a questionnaire regarding BNT162b2 COVID-19 vaccine adverse reactions following the administration of the two vaccine doses. The study population characteristics are described in Table 1.

**Figure 1:**
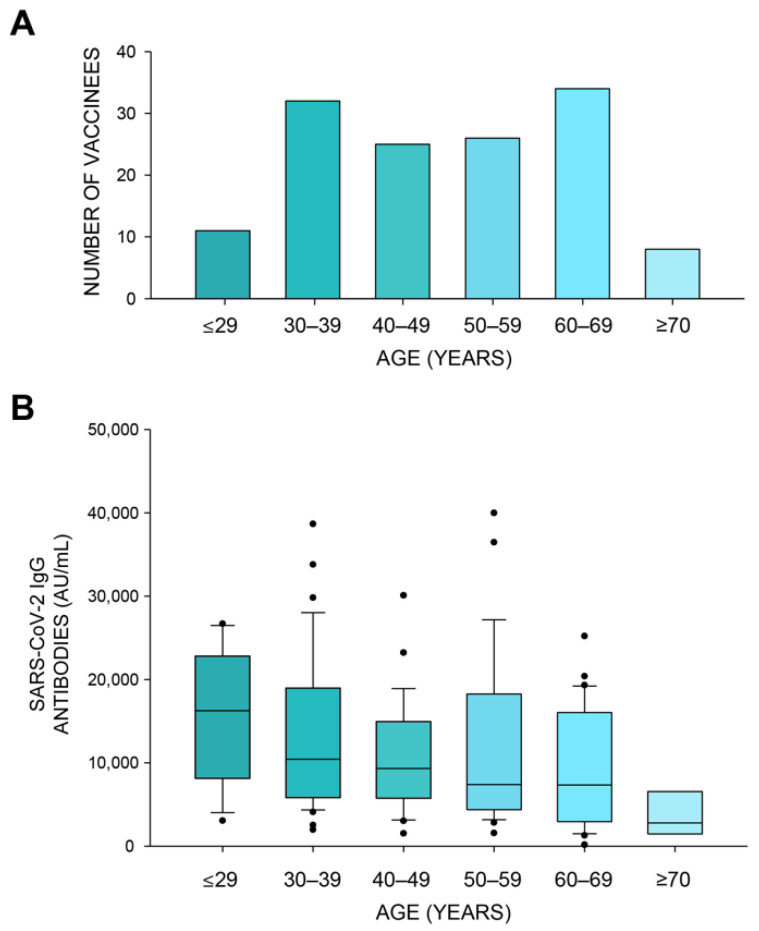

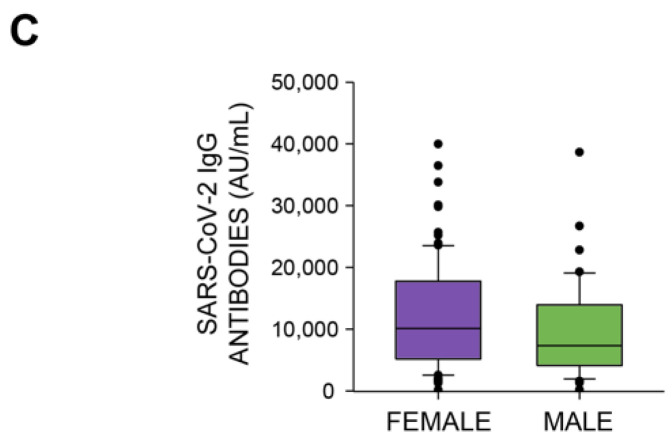
Antibody response following the BNT162b2 vaccine was greater in younger vaccinees. (**A**) Age distribution of study participants. (**B**) SARS-CoV-2 IgG levels across different age groups (Kruskal–Wallis One Way Analysis of Variance on Ranks: *p* < 0.01). (**C**) SARS-CoV-2 IgG levels in females and males (Mann–Whitney U test: *p* > 0.05). A positive response is defined as ≥50 AU/mL.

As was previously reported [10], the antibody response was found to be lower among older vaccinees (Kruskal–Wallis One Way Analysis of Variance on Ranks: *p* < 0.01, Figure 1A,B). No significant sex difference was observed in antibody levels (Mann–Whitney U test: *p* > 0.05, Figure 1C).

**Figure 2:**
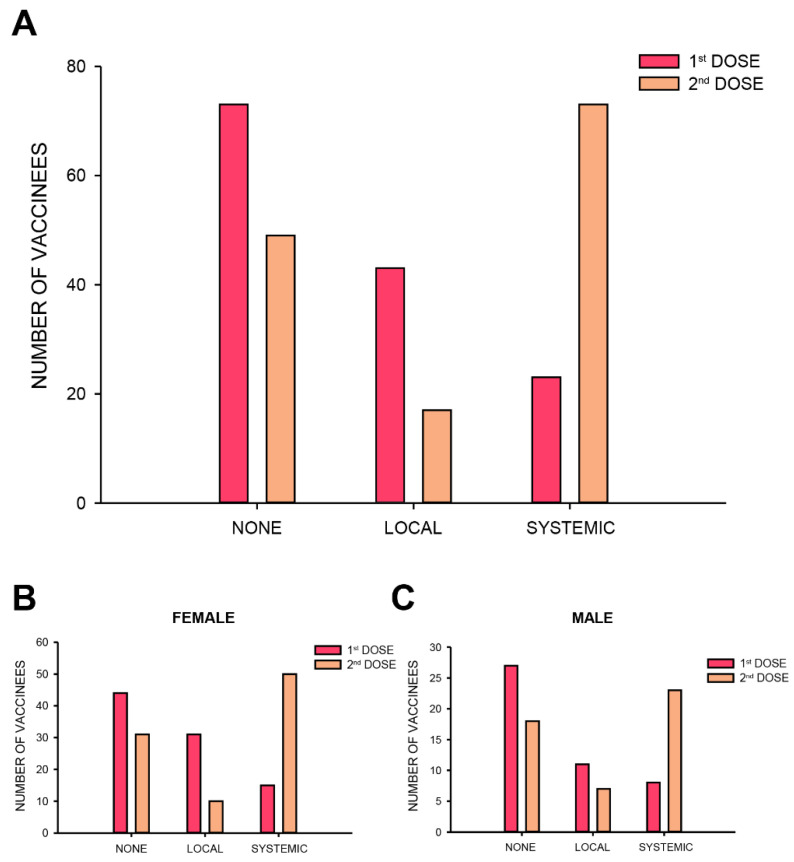
Adverse reactions shift from no/local reactions to systemic following the 2nd vaccine dose. (**A**) The distribution of the various types of reactions- none, local or systemic, following each vaccination dose (Chi-square, *p* < 0.001). (**B**,**C**) The majority of adverse reactions following the 2nd vaccine dose were systemic for both females (Chi-square, *p* < 0.001) and males (Chi-square, *p* < 0.01).

Participants reported more systemic adverse reactions following the second dose, as opposed to local or no adverse reactions following the first dose (Chi-square, *p* < 0.001, Figure 2A). These results did not change when accounting for sex (Chi-square, *p* < 0.001 and *p* < 0.01 for females and males, respectively, Figure 2B,C).

**Figure 3:**
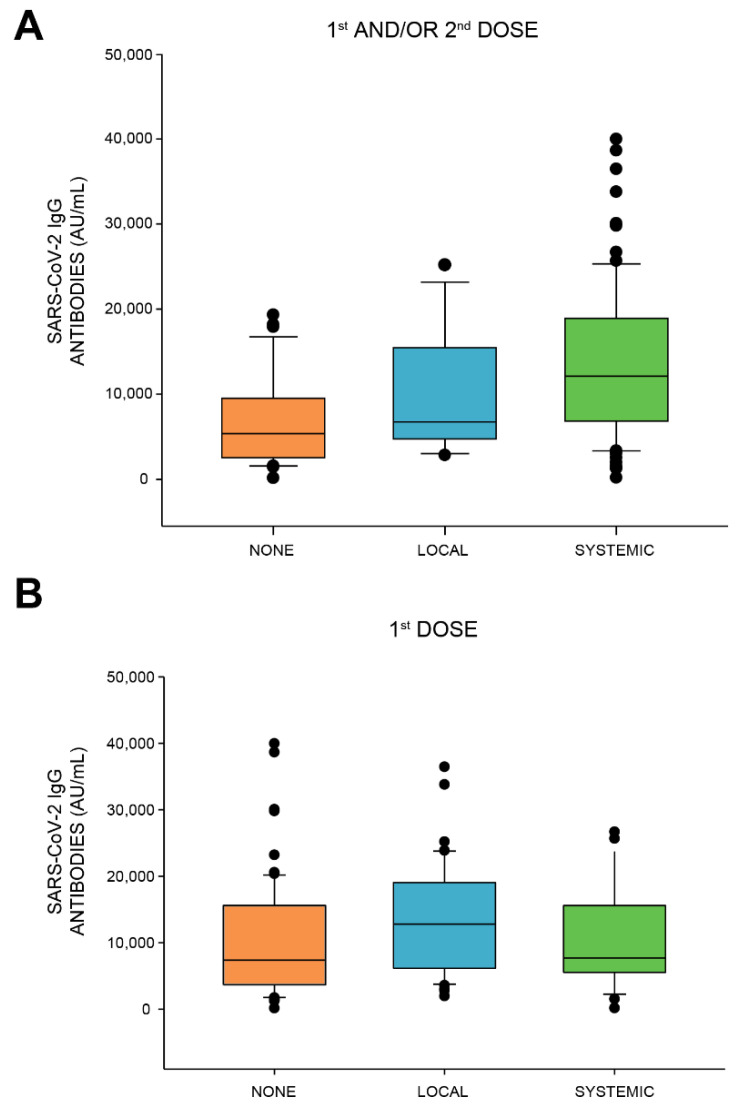

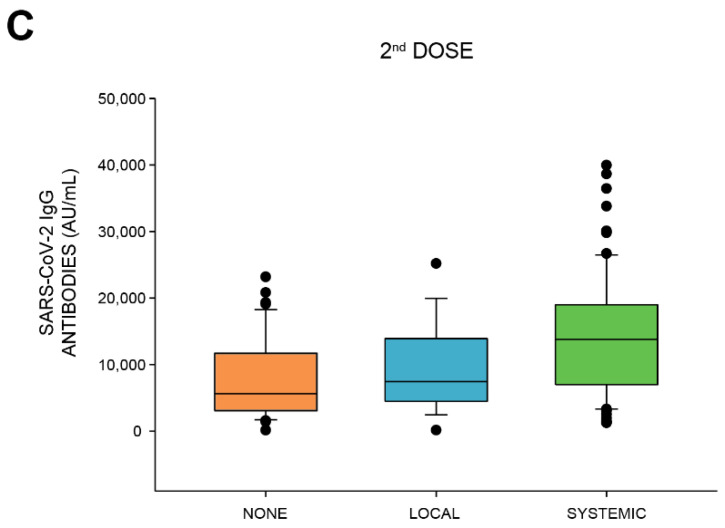
Association between SARS-CoV-2 IgG antibody response and adverse reactions. SARS-CoV-2 IgG levels in vaccinees who experienced no adverse reactions, isolated local adverse reactions, or any systemic adverse reaction (**A**) following either dose (Kruskal–Wallis One Way Analysis of Variance on Ranks: *p* < 0.001), (**B**) the first dose (Kruskal–Wallis One Way Analysis of Variance on Ranks: *p* < 0.05) or (**C**) the second dose (Kruskal–Wallis One Way Analysis of Variance on Ranks: *p* < 0.001). A positive response is defined as ≥50 AU/mL.

All of the 136 healthcare workers had a positive antibody response, defined as ≥50 AU/mL. The median antibody level in the cohort was 8879.5 AU/mL (interquartile range 4814.25–16,237.5). We found that vaccinees who experienced systemic adverse reactions (categorized according to Polack et al. [10], where injection site pain, redness, and swelling were classified as local, and any other adverse reactions classified as systemic) had significantly higher antibody levels than those who did not experience any adverse reactions (a median of 12,119 AU/mL and 5358 AU/mL, respectively, Kruskal–Wallis One Way Analysis of Variance on Ranks: *p* < 0.001, shown in Figure 3A). We also observed a higher antibody response level in favor of those who experienced any systemic adverse reaction over those who experienced solely local adverse reactions, albeit not significant (12,119 AU/mL and 6737 AU/mL, respectively). When comparing these results in relation to adverse reactions following each dose, we saw a significant correlation between higher antibody levels and local adverse reactions following the first dose, and between higher antibody levels and systemic adverse reactions following the second dose (Kruskal–Wallis One Way Analysis of Variance on Ranks: *p* < 0.05 and *p* < 0.001, Figure 3B,C).

## 4. Discussion

Here, we demonstrate for the first time a clear correlation between adverse reactions and antibody levels. Recent studies showed that higher antibody levels are correlated with protection from symptomatic SARS-CoV-2 infection [11,12,13]. Therefore, our findings indicate that non-serious adverse reactions may be predictive of a favorable anti-COVID-19 protective effect. Our study has some limitations, the first being the relatively small number of participants and their homogeneity. We did not perform baseline anti-spike antibody and/or anti-nucleocapsid antibody testing, therefore, we cannot unequivocally exclude the possibility that the serological response results may reflect an additive effect to a previously undetected infection in some subjects. Having said that, all study participants are previously COVID-19 naïve based on clinical diagnosis and the extensive routine SARS-CoV-2 RT-PCR surveillance according to the stringent policy applied to healthcare workers in Israel. Only one individual among the questionnaire responders was previously diagnosed with a COVID-19 infection and was excluded. In addition, no quantitative analysis of neutralizing antibodies was performed, however, several studies showed a positive correlation between IgG against the receptor-binding domain (RBD) and neutralizing antibody titers [14,15].

The waning of antibody levels over several months, accompanied by the surge of COVID-19 breakthrough infections [11], suggests a possible need for a 3rd dose. Indeed, such a program was recently initiated in Israel, where the majority of the population was vaccinated early upon the availability of the BNT162b2 vaccine. Recent data indicate that a booster dose rapidly and significantly increases antibody levels [16]. It might be suggested that those individuals that did not develop any adverse reaction following the first two vaccine doses should be prioritized and encouraged to receive a 3rd dose.

Although the use of mRNA for vaccination and other therapies was suggested years ago, a major limitation to the therapeutic use of mRNA was the excessive activation of innate immune response mediated by cytoplasmic RNA sensors resulting in enhanced cytokine production [17,18]. A seminal work demonstrated that the use of modified RNA nucleotides significantly decreased the inadvertent and harmful activation of the immune system and paved the way to the therapeutic use of mRNA [19]. While the use of modified nucleotides-based mRNA decreased the undesired activation of the immune system, the obliteration of the immune response is not complete. There is residual innate immunity activation, mainly through interferon (IFN) response [17]. This turned out to be an additional advantage of mRNA vaccines, which, in contrast to classical vaccines, do not require adjuvant additions.

Recent studies show that the most common reasons for hesitancy, specifically among ethnic minority groups who suffer from higher rates of COVID-19 related hospitalization and death [20,21], are concerns about side effects and long-term complications [22]. While we cannot yet provide evidence regarding the lack of long-term effects, our findings can help reduce vaccination hesitancy related to non-serious adverse events by reassuring that such reactions are to be expected and are correlated with a significant antibody response.

## Figures and Tables

**Table 1:** Study population characteristics.

Characteristic	*n* = 136
Age	
Mean (SD)—years	49.09 (13.92)
Female sex—no. (%)	90 (66.18)
Median antibody level (IQR)—AU/mL	8879.5 (4814.25–16,237.5)
Time between 2nd dose and blood drawn	
Mean (SD)—days	32.4 (4.84)
Adverse reactions following the 1st dose–no. (%)	
None	71 (52.2)
Local	42 (30.9)
Systemic	23 (16.9)
Adverse reactions following the 2nd dose—no. (%)	
None	48 (35.3)
Local	17 (12.5)
Systemic	71 (52.2)

## Data Availability

The data presented in this study are available on request from the corresponding author due to privacy restrictions.
